# Passive immunization with an extended half-life monoclonal antibody protects Rhesus macaques against aerosolized ricin toxin

**DOI:** 10.1038/s41541-020-0162-0

**Published:** 2020-02-13

**Authors:** Chad J. Roy, Greta Van Slyke, Dylan Ehrbar, Zachary A. Bornholdt, Miles B. Brennan, Lioudmila Campbell, Michelle Chen, Do Kim, Neil Mlakar, Kevin J. Whaley, Jeffrey W. Froude, Fernando J Torres-Velez, Ellen Vitetta, Peter J. Didier, Lara Doyle-Meyers, Larry Zeitlin, Nicholas J. Mantis

**Affiliations:** 1 Tulane National Primate Research Center, Covington, LA 70433 USA; 2 Division of Infectious Disease, Wadsworth Center, New York State Department of Health, Albany, NY 12208 USA; 3 Mapp Biopharmaceutical, Inc, San Diego, CA 92121 USA; 4 Clinical Pharmacology Branch, Walter Reed Institute of Research, 503 Robert Grant Ave, Silver Spring, MD 20910 USA; 5 Departments of Immunology and Microbiology, University of Texas Southwestern Medical Center, Dallas, TX 75390 USA; 6 Present Address: Vaccines and Therapeutics Division, Defense Threat Reduction Agency, 8725 John J. Kingman Rd., Fort Belvoir, VA 22060 USA

**Keywords:** Antibodies, Bacterial toxins, Vaccines

## Abstract

Inhalation of ricin toxin (RT), a Category B biothreat agent, provokes an acute respiratory distress syndrome marked by pro-inflammatory cytokine and chemokine production, neutrophilic exudate, and pulmonary edema. The severity of RT exposure is attributed to the tropism of the toxin’s B subunit (RTB) for alveolar macrophages and airway epithelial cells, coupled with the extraordinarily potent ribosome-inactivating properties of the toxin’s enzymatic subunit (RTA). While there are currently no vaccines or treatments approved to prevent RT intoxication, we recently described a humanized anti-RTA IgG_1_ MAb, huPB10, that was able to rescue non-human primates (NHPs) from lethal dose RT aerosol challenge if administered by intravenous (IV) infusion within hours of toxin exposure. We have now engineered an extended serum half-life variant of that MAb, huPB10-LS, and evaluated it as a pre-exposure prophylactic. Five Rhesus macaques that received a single intravenous infusion (25 mg/kg) of huPB10-LS survived a lethal dose aerosol RT challenge 28 days later, whereas three control animals succumbed to RT intoxication within 48 h. The huPB10-LS treated animals remained clinically normal in the hours and days following toxin insult, suggesting that pre-existing antibody levels were sufficient to neutralize RT locally. Moreover, pro-inflammatory markers in sera and BAL fluids collected 24 h following RT challenge were significantly dampened in huPB10-LS treated animals, as compared to controls. Finally, we found that all five surviving animals, within days after RT exposure, had anti-RT serum IgG titers against epitopes other than huPB10-LS, indicative of active immunization by residual RT and/or RT-immune complexes.

## Introduction

The Centers for Disease Control and Prevention (CDC), along with other U.S. government and international agencies, recognize that the deliberate release of biological toxins and highly infectious agents into the public sphere remains a threat to both military and civilian personnel.^1,2^ Ricin toxin (RT), for example, was recently ranked by NATO’s Biomedical Advisory Council as a high potential threat because of its accessibility, stability, and extreme toxicity, especially by inhalation.^3^ RT is a ~65 kDa glycoprotein from the castor bean plant (*Ricinus communis*) that kills mammalian cells with remarkable efficiency through the coordinated activity of two subunits, RTA and RTB.^4^ RTB, a galactose-specific lectin, mediates the endocytosis and intracellular trafficking of RTA from the plasma membrane to the endoplasmic reticulum (ER), by way of the trans Golgi network (TGN). Within the lumen of the ER, RTA is liberated from RTB and then translocated into the cytoplasm where it irreversibly inactivates ribosomes by depurination of a conserved adenosine moiety within the sarcin/ricin loop of 28 S rRNA.^5,6^ Ribosome arrest triggers the so-called ribotoxic-stress response (RSR), which, in turn, activates stress activated protein kinase pathways and programmed cell death (PCD).

The full cytotoxic and inflammatory potential of RT are especially apparent in the lung following aerosol exposure. Following inhalation, RT exposure manifests as hemorrhage, inflammatory exudate, and edema.^7,8^ A precipitous decline in alveolar macrophages (AMs) occurs within hours and is accompanied by a corresponding influx of polymorphonuclear (PMN) cells.^9^ Disease severity and lung permeability track with the increase of pro-inflammatory cytokines like IL-6, IL-1, and tumor necrosis factor alpha (TNF-α) in serum and bronchoalveolar lavage (BAL) fluids.^10–12^ Local damage is further exacerbated by TNF-α and its family members like TRAIL, which render lung epithelial cells hyper-sensitive to the effects of RT, as evidenced by augmented (>300 fold) secretion of IL-6 and other pro-inflammatory cytokines, as well as a lower threshold to elicit PCD.^13^ This positive feedback loop likely contributes to the rapid onset of acute lung injury that is observed in Rhesus macaques following RT aerosol exposure.^8,11^

Given the rapid onset of RT toxicity following aerosol exposure, the opportunity to intervene and reverse the effects of lethal dose RT exposure is likely to be on the order of hours. As a case in point, we recently reported that huPB10, a humanized monoclonal antibody (MAb) directed against an immunodominant epitope near RTA’s active site, was able to rescue Rhesus macaques from a 3 × LD_50_ RT aerosol challenge when administered ~4–6 h after toxin exposure.^11^ The MAb presumably intercepted extracellular (free or surface-bound) ricin in the lung environment before it was internalized by target cells, thereby preventing macrophage and epithelial cell intoxication. HuPB10 intervention also dramatically blunted the local and systemic pro-inflammatory cytokine and chemokine responses normally associated with RT exposure, with the most apparent effect being on IL-6.

While the demonstration that a single huPB10 infusion was able to rescue Rhesus macaques from the lethal effects of RT justifies the pursuit of a variety of post-exposure therapies, the relatively short therapeutic window associated with lethal dose RT prompted us to explore the use of huPB10 as a pre-exposure prophylactic (PrEP). The benefits of passive immunization strategies are well recognized, particularly within the realm of biodefense where access to immediate immunity is critical.^14^ In the case of huPB10, proof of concept pre-exposure prophylaxis studies have already been conducted in mice. In the mouse model, full protection against 10 × LD_50_ intranasal RT challenge was achieved when serum levels of huPB10 were ≥20 μg/ml, thereby providing us with a rough benchmark for NHP studies.^15^ HuPB10 production has already been shown to be scalable in both plant- and mammalian-based platforms^11^ and was therefore chosen as a candidate to pursue as a PrEP.

## Results

### Recombinant huPB10 IgG variants with extended serum half-lives in NHPs

In an effort to enhance the serum half-life of huPB10 before embarking on PrEP studies in NHPs, we engineered variants of huPB10 with YTE and LS modifications of the Fc region that enhance interaction with FcRn.^16,17^ The YTE (M252Y, S254T, T256E) and LS (M428L, N434S) IgG_1_-variant constructs were each evaluated for binding affinities and RT-neutralizing activity in vitro and in passive protection studies in a mouse model of systemic RT challenge. Because the YTE and LS variant performed equivalently in vitro, the choice to advance the LS variant for NHP studies was based on the demonstration by others that LS variants have slightly longer half-lives than YTE variants in some experimental settings.^17^ HuPB10 and huPB10-LS versions were expressed in a non-fucosylating CHOK1-AF cell line and purified via Protein A affinity chromatography.

The pharmacokinetic (PK) properties of huPB10 and huPB10-LS were determined in Rhesus monkeys (*Macaca mulatta*). Animals (*n* = 9; average weight = 8.0 ± 2.01 kg) were dosed intravenously (IV) with 10 mg/kg and monitored over a period of 60 days. The serum half-life (*T*_1/2_) of the huPB10-LS formulation following IV delivery was more than double the huPB10 IgG1 version (28.5 versus 12.4 days) (Supplementary Fig. 1). The *T*_1/2_ values of huPB10-LS was similar when the MAb was administered intramuscularly (21.8 days) or subcutaneously (23.7 days). In fact, the *T*_1/2_ values of huPB10-LS were greater than huPB10 by all three routes of administration.

### HuPB10-LS IgG pre-exposure prophylaxis protects macaques from aerosol RT exposure

To assess the potential of huPB10-LS to afford passive immunity to aerosolized RT exposure, a group of five Rhesus macaques received a single IV injection of huPB10-LS (25 mg/kg) on study day −28 (Table 1; Supplementary Fig. 2). Three control animals received an IV injection of an anti-SEB IgG_1_ (Ig121), kindly provided by Dr. Javad Aman (Integrated Biotherapeutics).^18^ The serum concentrations of huPB10-LS IgG1 in the experimental animals on the day of challenge ranged between 113 and 315 μg/ml and are consistent with expected dosing regimen (Table 1).

**Table 1:** Experimental animals and outcome of RT aerosol challenge.

					huPB10 (μg/mL)^a^			
Treatment	ID	kg	RT^b^	LD_50_^c^	serum	BAL	TTD^d^	live/total
Ig121	KA06	10.32	18.4	3	–	–	46	
	KN88	6.67	21.3	3.5	–	–	32	
	LE35	7.03	27.0	4.5	–	–	30	0/3
huPB10-LS	LC63	6.8	36.8	6.1	315.2	0.19	–	
	LA76	6.6	35.9	5.9	177.7	0.38	–	
	LA17	5.6	34.4	5.7	113.4	0.37	–	
	LH54	5.12	26.1	4.4	213.3	0.32	–	
	LI64	4.57	37.1	6.2	244.9	0.40	–	5/5

^a^huPB10-LS (μg/mL) in serum collected prior to challenge and BAL collected on day −7.

^b^Ricin toxin (RT) dose (μg/kg) received per animal.

^c^LD_50_ equivalent.

^d^Time to death (TTD).

**Fig. 1:**
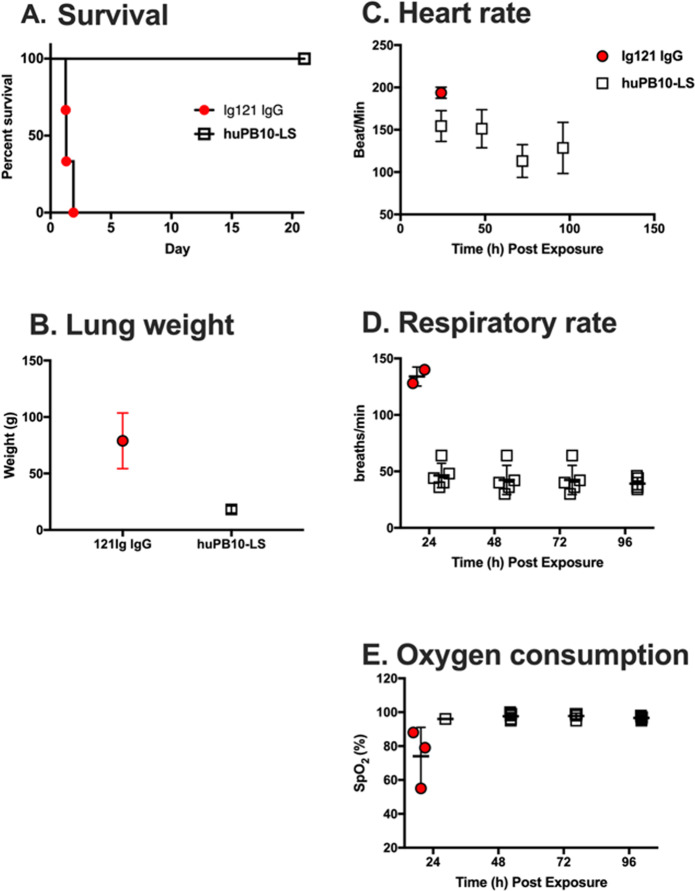
Prophylactic administration of huPB10-LS IgG protects Rhesus macaques from lethal dose aerosolized RT. Groups of Rhesus macaques received Ig121 IgG (*n* = 3) or huPB10-LS IgG on day −28 and then challenged with RT aerosol, as described in the text. The experiment was terminated on day + 21 at which time surviving animals were euthanized and subjected to necropsy. **a** Kaplan-Meier survival plot. All five monkeys treated with huPB10-LS IgG survived RT exposure. **b** Wet lung weight (both lobes; average ± SD) at necropsy. **c**–**e** Heart rates, respiratory rates and oxygen consumption in two groups of animals at indicated time (hours) post-RT challenge.

On study day 0, all eight animals were exposed to RT by small particle aerosol at a target dose of 18 µg/kg or the equivalent of ~3 × LD_50_ (Table 1). On average, the control group received 22.2 ± 4.4 µg/kg RT (3.8 ± 0.7 × LD_50_), while the huPB10-LS experimental group received 34.1 ± 4.5 µg/kg (5.8 ± 0.7 × LD_50_); collective RT challenge dose across all animals was 29.6 ± 7.4 (5.1 ± 1.2 × LD_50_). As expected, all three control animals succumbed to ricin intoxication within a 30–48 h period (Table 1; Fig. 1a). In contrast, all five of the animals pretreated with huPB10-LS survived RT challenge. In the immediate period following toxin exposure, the control animals demonstrated elevated heart and respiratory rates and reduced oxygen consumption, as compared to the huPB10-LS group of animals, whose general physiology was considered normal. Blood chemistry analysis revealed distinct differences between the control (Ig121-treated) and huPB10-LS treated animals following ricin challenge (Supplementary Fig. 3). Most notably was the elevated level of total white blood cells in the control animals (45.1 ± 16.0 × 10^3^/mL), as compared to either pre-challenge counts of all animals (7.8 ± 1.2 × 10^3^/mL) and the huPB10-LS treated group of animals (18.8 ± 7.8 × 10^3^/mL).

At necropsy, the wet weight of the lungs of the control animals ranged from 50 to 100 g, as compared ~30–40 g in normal animals of the same body weight, based on historical data at the Tulane National Primate Research Center (TNPRC). The wet weights of the lungs of the huPB10-LS animals at necropsy following euthanasia on study day 21 were within normal range (Fig. 1b; Supplementary Fig. 4). Gross examination of the lungs from control animals following RT challenge revealed coalescing hemorrhage with frothy exudate marked by fibrin in lung parenchyma, as we have reported previously.^11^ There was evidence of fibrinosuppurative bronchointerstitial pneumonia with pulmonary edema and bronchial epithelial necrosis, with severe fibrinosuppurative lymphadenitis in the bronchial lymph nodes. Histologically, the lungs of the control animals had all the hallmarks of RT-induced injury; edema, corresponding hemorrhage, and numerous PMN cells. In contrast, the lumina of airways from huPB10-LS treated animals were clear and the epithelial surfaces devoid of inflammatory cells. Most tissues were negative for the eosinophil major basic protein (MBP), as determined by IHC. In two huPB10-LS treated animals, there was evidence of minimal infiltration of airways epithelia with equal proportions of neutrophils and eosinophils (Supplementary Figs 5–6).

### Analysis of inflammatory markers in serum and BAL fluids following RT exposure

**Fig. 2:**
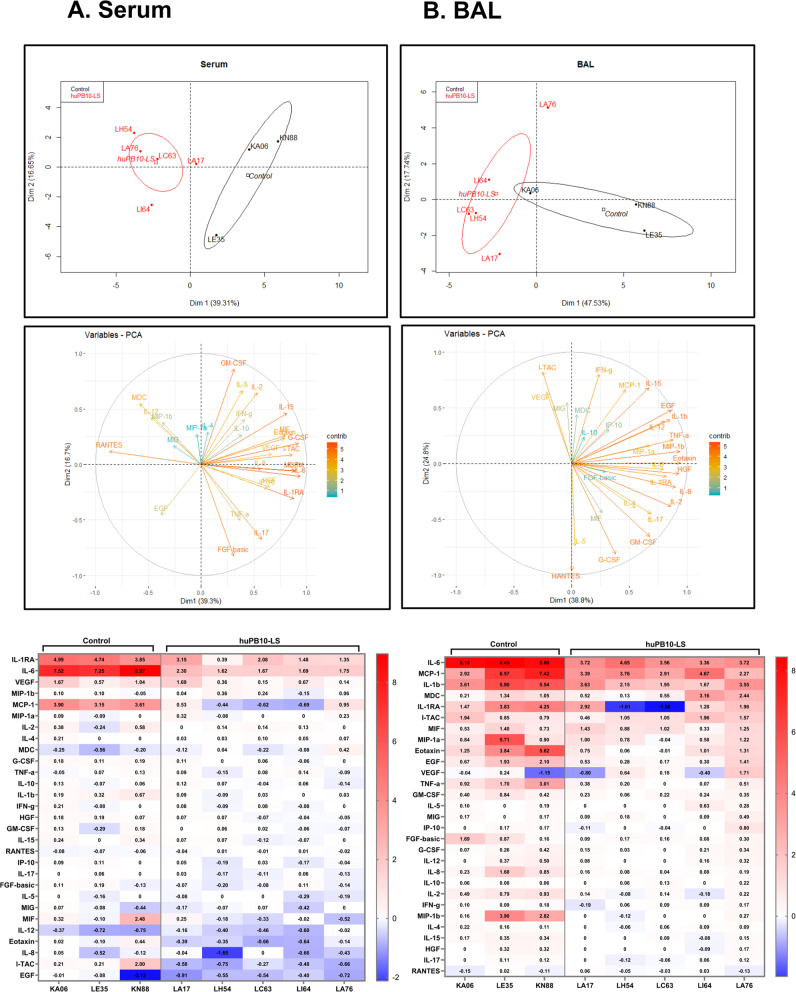
Pre-existing huPB10-LS IgG dampens local inflammatory responses associated with aerosol RT exposure. Serum samples (**a**) and BAL fluids (**b**) collected from Ig121 IgG- and huPB10-LS IgG-treated animals before and 24 h after RT exposure were subjected to 29-plex Luminex analysis, as described in the Materials and Methods. Top: Scatter plots of first 2 principal components of log_2_-transformed cytokine fold changes from all animals included in the study, calculated used singular value decomposition. Each dot represents an individual monkey in the Ig121 (black) and huPB10-LS (red) groups. Ellipses represent 95% confidence intervals around group mean. Middle: Eigenvectors are colored to show the percent contribution of each variable to the principal components. Bottom: Heatmaps presenting log_2_-fold change in cytokines, chemokines, and growth factors (y-axis) in the sera and BAL fluids between samples collected experimental day +1, to day −1 (serum) and −7 days (BAL fluids). Individual animal identifiers are shown on the x-axis and correspond to Table 1. Fold increase in values are shown in red, while fold decreases are in blue.

Serum samples and BAL fluids collected from the study animals (three control and five huPB10-LS treated) before and one day after RT challenge were analyzed by 29-plex Luminex array to assess the impact of pre-existing huPB10-LS on systemic and local inflammatory responses (Fig. 2). Principle component analysis (PCA) of RT-induced changes in serum cytokines successfully segregated the control and the experimental animals into non-overlapping clusters (Fig. 2). The cytokines IL-6, G-CSF, IL-15, IL-1RA, and GM-CSF, listed in order of influence, were the largest contributors to the first two principle components (Fig. 2; Supplementary Data 1).

PCA of cytokine expression in the BAL fluids resulted in slightly overlapping clusters for the two groups, due to skewing by a single macaque (KA06) in the control group (Table 1; Fig. 2). KA06 was the heaviest animal in the experiment (10.32 kg) and survived the longest (46 h post-challenge). By chance, it also received the lowest dose of RT of any animal in the study (18.4 µg/kg). Cytokine responses to RT challenge were muted in this animal relative to the other controls, as evidenced by the smallest fold changes in MCP-1, IL-1b, MDC, and IL-1RA, along with others in the BAL fluid. Otherwise, EGF, IL-1b, MIP-1b, eotaxin, and HGF were the main drivers of variation in the BAL fluid dataset (Fig. 2; Supplementary Data 1).

The effect of huPB10-LS on overall cytokine levels was remarkable. In sera, the concentration of IL-6 increased ~1800-fold in controls, but only ~16-fold in the macaques pretreated with huPB10-LS (Fig. 2). Similarly, concentrations of IL-1RA and MCP-1 were elevated ~29-fold and 6.6-fold, respectively, in the control animals, but only 5-fold and not elevated in the case of huPB10-LS treated monkeys. The trend was similar in the BAL, although the overall degree of dampening by huPB10-LS was less than observed in sera (Fig. 2). As a case in point, the concentration of IL-6 was elevated at least ~1000-fold in the BAL fluids of the controls (pre versus post RT exposure), and 45-fold in huPB10-LS group. These results demonstrate that pre-existing levels of huPB10-LS in sera and/or BAL fluids significantly dampen both local and systemic inflammatory responses elicited following inhalation of RT.

### de novo RT-antibody responses in huPB10-LS treated Rhesus macaques following RT challenge

**Fig. 3:**
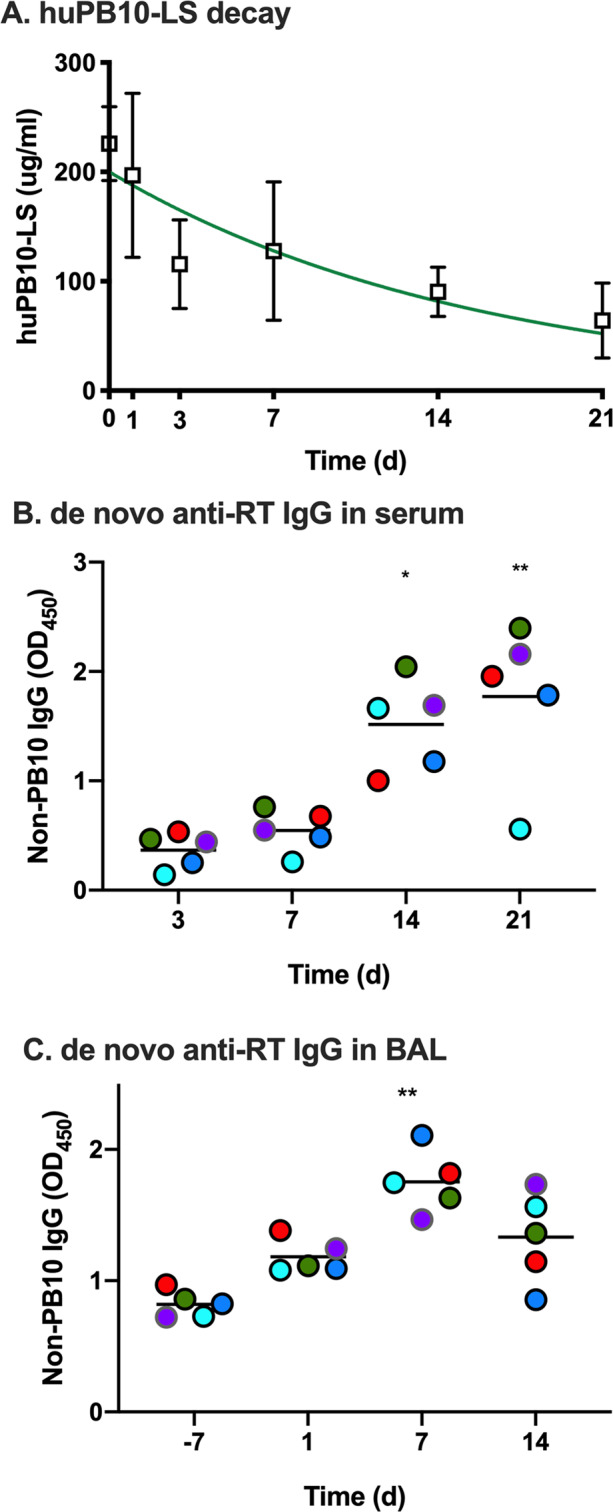
De novo appearance of ricin-specific antibodies in serum and BAL fluids in huPB10-LS treated animals following RT exposure. **a** Average (with SEM) serum huPB10-LS IgG1 concentrations at timepoints immediately before (day 0; Table 1) and after (days 1,3,7,14, 21) RT challenge. **b**, **c** Appearance of non-PB10, RT-specific IgG antibodies in (**b**) serum and (**c**) BAL fluids in the experimental group of Rhesus macaques following RT challenge, as determined by a PB10 capture ELISA (see Materials and Methods). Each colored circle represents a single animal. The horizontal bar represents the mean values at each timepoint with asterisks indicating values that were significantly different from baseline (day 0) levels (*p < 0.05; **p < 0.005), as determined by Dunn’s multiple comparisons tests. (**b**) Friedman statistic = 14.04, *p*-value < 0.0001. **c** Friedman statistic = 12.12, *p*-value = 0.0014.

**Fig. 4:**
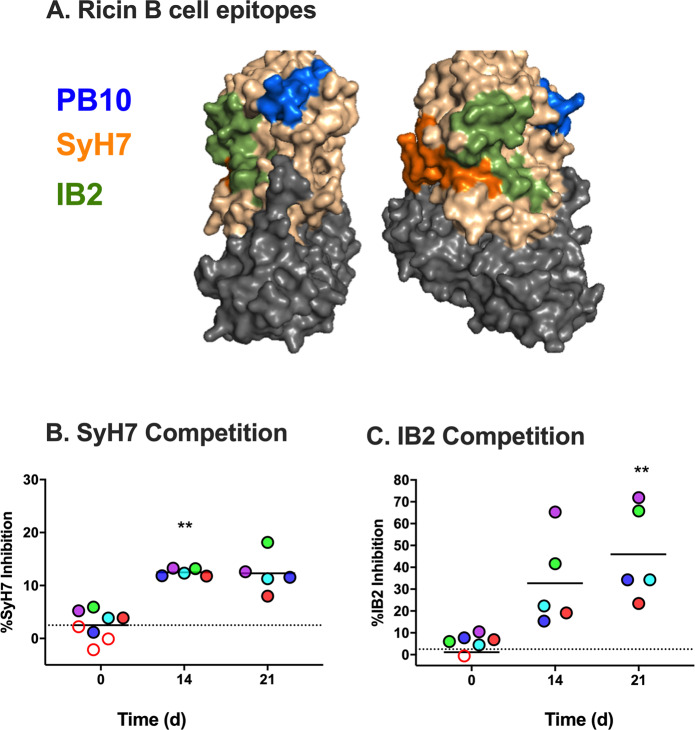
Epitope reactivity of RT-specific serum antibodies in Rhesus macaques following RT exposure. **a** PyMol surface representation of RT (PDB ID: 2AAI) with RTA (sand) and RTB (charcoal) depicted. The PB10 (blue), SyH7 (orange) and IB2 (green) epitopes are highlighted. **b**, **c** Modified competition ELISA in which microtiter plates were coated with capture MAbs SyH7 or IB2, and then probed with biotin-labeled RT in the presence of sera (1:25 dilution) from control (open circles) or huPB10-LS IgG-treated animals (colored circles). The horizontal bar represents the mean at each timepoint with asterisks indicating values that were significantly different from baseline (day 0) levels (*p < 0.05; **p < 0.005), as determined by Dunn’s multiple comparisons tests. **b** Friedman statistic = 8.400, *p*-value = 0.0085. **c** Friedman statistic = 10.00, *p*-value = 0.0008.

Following RT challenge, levels of huPB10-LS in sera of the Rhesus macaques declined with the expected half-life steadily over the remaining three weeks of the study (Fig. 3). However, concomitant with the decline in circulating huPB10-LS levels (as detected using a peptide-specific ELISA), all five surviving Rhesus macaques experienced an onset of RT-specific IgG antibodies in serum and BAL fluids, as revealed by a mouse PB10 capture ELISA (Fig. 3b, c; Supplementary Fig. 7). To examine the nature of the de novo anti-RT polyclonal antibody response in more detail, serum samples from all five Rhesus macaques collected on day 21 post RT challenge were depleted of huPB10-LS using a peptide affinity column (Supplementary Fig. 8). The resulting antisera were then examined in a competitive ELISA for reactivity against two known immunodominant epitopes on RTA, represented by MAbs SyH7 and IB2 (Fig. 4a).^19^ Indeed, all five serum samples reacted to some degree with immunodominant epitopes represented by SyH7 and IB2 (Fig. 4b, c). These results are evidence that all five huPB10-LS treated Rhesus macaques mounted an active anti-RT-immune response in the days following RT exposure, most likely induced in response to low level residual RT or RT-immune complexes in the lungs.

## Discussion

Among the CDC’s list Select Agents and Toxins, none is a more potent provocateur of pulmonary inflammation and acute lung injury than RT. The severe damage to the respiratory mucosa following RT exposure is the result of direct cytotoxic effects on AMs and airway epithelial cells, combined with a profuse pro-inflammatory response driven by IL-6, IL-1, and TNF-α.^7,8^ In this report we demonstrate for that pre-exposure infusion with a single MAb at a dose of 25 mg/kg is sufficient to protect NHPs against a lethal dose RT aerosol challenge one month later. The five Rhesus macaques that received huPB10-LS IgG had slightly elevated pro-inflammatory cytokine levels in their sera and BAL fluids immediately following RT aerosol exposure, but were otherwise normal in the days and weeks following challenge. In fact, by all metrics, huPB10-LS prophylaxis afforded the same degree of immunity to Rhesus macaques as was achieved through active vaccination with RiVax, an RTA-based subunit vaccine^20^. This raises the prospect that a single MAb-based drug product can afford both immediate and long-lasting (e.g., months) immunity to a notorious biothreat agent.^14^

We found that the LS modification (M428L, N434S) more than doubled the serum half-life of huPB10-LS from 12.3 days to 28.4 days in Rhesus macaques. As both the LS and YTE mutations were optimized for binding to human FcRn, the serum half-life for these mAbs in humans is even more extended, with a *T*_1/2_ range of 63 to 117 days in the clinical trials described to date.^21–24^ Consequently, a single infusion of huPB10-LS at 25 mg/kg yielded an average of 212 μg/ml of MAb in serum 28 days later, at the time of challenge. While the minimal threshold of huPB10-LS required to protect Rhesus macaques from RT aerosol challenge has not been established, in the mouse model the estimate is ~20 μg/ml is required for protection against 10 × LD_50_ challenge.^15^ If this threshold were to apply to Rhesus macaques (or humans), then the single infusion of huPB10-LS IgG1 at the doses used here would theoretically provide up to four months’ worth of protection.

We postulate that low levels of pre-existing huPB10-LS protect the lung from RT by binding to free and surface-bound toxin immediately following aerosol exposure. Previous studies from our group suggest that RT remains accessible to antibodies following aerosol exposure for at least 4 h.^11^ HuPB10-LS likely also scavenges RT that gains access to interstitial fluids. At that point, antibody-toxin immune complexes could meet several fates, including mechanical clearance from the airways^25^ or uptake via non-professional or processional antigen cells (APCs) in the lung mucosa. For example, in the laboratory, human epithelial cell lines (e.g., HeLa cells) internalize PB10-RT-immune complexes and route them to the lysosome for degradation.^26^ Whether lung epithelial cells perform a similar function in vivo is unknown. AMs also have the capacity to sample antibody-antigen complexes and, in the case of ricin, could do so through FcR-mediated uptake and/or the mannose receptor (CD206), which recognizes RT’s three N-linked mannose side chains.^27,28^ Finally, it is possible that RT-antibody complexes are sampled by local dendritic cells (DCs), and then trafficked to regional lymph nodes where presentation to B cells can occur.^29^ In data not presented in this manuscript, we have observed the onset of a de novo RT-neutralizing antibody response induced within 14 days in huPB10-LS-treated Rhesus macaques following RT exposure. This phenomenon is reminiscent of the so-called “vaccinal effect,” which refers to the ability of immune complexes to act as self-adjuvanted immune stimulators.^30–32^ This exact phenomenon was observed decades ago by Lemely and Wright when mice were passively immunized with a PB10-like MAb called UNIVAX70 and then challenged with RT by the subcutaneous route.^33^ While this effect is not necessarily an added benefit for an agent like RT, it could certainly have important applications in reoccurring diseases like flu or even pneumonia.

## Methods

### Ricin toxin (RT)

The purified RT from castor beans (*Ricinus communis*) used in this study was from the same lot used in previous Rhesus macaques challenge studies.^11,20^

### MAbs

HuPB10-LS was expressed using a non-fucosylating CHOK1-AF host cell line. Cells were grown in fed batch culture in 2.5 L shake flasks using BalanCD CHO Growth A media (FUJIFILM Irvine Scientific, Santa Ana, CA) and Efficient Feed B (ThermoFisher Scientific, Waltham, MA). The conditioned media were collected, filtered, and stored at −20C prior to PrA purification. The conditioned media was thawed and loaded onto a MabSelect SuRe LX Protein A column, washed with PBS, then eluted using 0.1 M Acetic Acid containing 0.2 M L-Arginine, pH ~2.9. The resulting eluate was neutralized with 2 M Tris base to pH ~7. The huPB10-LS MAb was further purified using ceramic hydroxyapatite (CHT) Type II, 40 µm, column (Bio-Rad, Hercules, CA). The CHT column removed the majority of the aggregates present after PrA purification. The neutralized post-Protein A eluate was diluted five-fold using Milli-Q water to reduce the conductivity to ≤ 6 mS/cm before loading onto the CHT column. The diluted sample was loaded, washed with 5 mM sodium phosphate, pH 6.8 wash buffer, then eluted using a linear gradient from 5 mM sodium phosphate to 250 mM sodium phosphate, pH 6.8 elution buffer over 30 column volumes. The linear gradient was held at ~28% when the UV_280_ reached the maximum absorbance to elute remaining antibody on the column. The MAb was then subjected to tangential flow filtration and buffer exchanged into formulation buffer (20 mM citrate, 10 mM glycine, 8% sucrose, 0.01% polysorbate 80, pH 5.5) and tested. The concentration of the formulated huPB10-LS drug substance was 22.1 mg/mL with 97% monomer via SE-HPLC and an endotoxin level of 0.055 EU/mg. Control animals received the anti-SEB IgG_1_ (Ig121) provided by Dr. Javad Aman (Integrated Biotherapeutics).

### Study designs and approval

The studies described involving Rhesus macaques (*Macaca mulatta*) were approved animal use protocols by the Institutional Animal Care and Use Committee (IACUC) at Tulane University, New Orleans, LA. Rhesus macaques were born and housed at the Tulane National Primate Research Center (Covington, LA), which is US Department of Agriculture-licensed and fully accredited by the Association for Assessment and Accreditation of Laboratory Animal Care (AAALAC). To establish the pharmacokinetic (PK) properties of huPB10 and huPB10-LS, animals (*n* = 9; average weight = 8.0 ± 2.01 kg) were dosed intravenously (IV), intramuscularly (IM) or subcutaneously (SC) with 10 mg/kg and monitored over a period of 60 days. For the RT challenge study, groups of animals received a single IV administration of huPB10 or (control antibody IgG121) by slow infusion at an individualized unit dose of 25 mg/kg on study Day −28. Treated animals were observed for signs of adverse reactions to the MAb during administration and throughout the anesthesia recovery period. Animals that were determined to be in respiratory distress and those that survived for 21 days after exposure to ricin were euthanized by an overdose of sodium pentobarbital, consistent with the recommendation of the American Veterinary Medical Association’s Panel on Euthanasia, and submitted for necropsy.

### RT challenge

Aerosolization, dosing and delivery of RT were performed on study Day 0.^20^ The LD_50_ of ricin is 5.8 µg/kg body weight and the target dose for this experiment was set at the equivalent of 3 × LD_50_s (≈18 µg/kg). The mean inhaled dose of ricin across all animals was 29.7 ± 7.4 µg/kg or 5.1 ± 1.2 LD_50_s. The schedule for collection of blood and BAL^34^ are shown in Fig. S2. Animals were euthanized upon completion of the experiment (study Day +21) or at any time during the experiment for humane reasons, as mandated by Tulane University’s IACUC. Euthanasia was achieved with an overdose of sodium pentobarbital, as per the recommendation of the American Veterinary Medical Association’s Panel on Euthanasia. All animals were subject to complete necropsy. After gross necropsy, tissues were collected in neutral buffered zinc-formalin solution (Z-Fix Concentrate, Anatach, Battle Creek, MI). Fixed tissues were embedded in paraffin and sectioned for IHC.^8^

### IHC

Tissue sections, 3–4 μm in thickness, were deparaffinized in CitriSolve (Decon Labs., King of Prussia, PA) and rehydrated by processing them through graded alcohol solutions. Antigen unmasking was accomplished by digesting tissue in 10 μg/ml of protein kinase for 15 min at room temperature (Fisher Scientific, Waltham, MA). Endogenous enzymes and non-specific background were blocked with Background Punisher (Biocare Medical, Pacheco, CA), followed by BLOXALL (Vector Laboratories, Burlingame, CA). The primary antibody (NBP1–42140; Novus Biologicals, Centennial, CO) was incubated on the tissue sections at a dilution of 1:100 for 1 h at room temperature. Subsequently, sections were sequentially incubated with an alkaline phosphatase-based detection polymer kit (MACH 4; Biocare Medical), and Warp Red (Biocare Medical). Sections were counterstained with Tacha’s hematoxylin (Biocare Medical) and mounted using a permanent mounting medium (EcoMount; Biocare Medical).

### Serum antibody analysis

For the PK study, an Octet RED96 based method was used for quantitation of huPB10 serum concentration. In order to quantitate the amount of huPB10 IgG MAb and -LS MAb variant in NHP serum samples, we created custom quantitative biosensors. Biotinylated *Ricinus communis* agglutinin II (Vector Laboratories, Cat: B-1095) was diluted to 50 μg/mL in 2× kinetics buffer and immobilized on High Precision Streptavidin (SAX) Biosensors (Pall ForteBio, Cat: 185117) for 3.5 h at room temperature without shaking. The ricin coated sensors were then run through a standard ForteBio dip and read quantitation method using each animal’s Day 0 serum sample as background subtraction and quantitated using an huPB10 IgG standard curve (40, 20, 10, 5, 2.5, 1.25, and 0.625 μg/ml diluted into 2x kinetics buffer). NHP samples were diluted in a 2:3 ratio of serum sample to 2x kinetics buffer to further reduce background. A 100x kinetics buffer stock was created from 10 mg of BSA, 20 µL of Tween-20, and 10 mL of PBS.

For ELISAs, Immulon 4HBX 96-well microtiter plates were coated with RT or E12 peptide corresponding to RTA residues 94–111 (HPDNQEDAEAITHLFTDV).^35^ Serum huPB10-LS IgG concentrations were determined by interpolation on a standard curve (huPB10 IgG1) using a nonlinear regression model and least squares fit in GraphPad Prism 8.2 (GraphPad Software, La Jolla California USA). HuPB10-LS IgG was depleted from day 21 serum samples using MicroLink™ (ThermoScientific) affinity column filtration, according to the manufacturer’s instructions. Columns were coupled with E12 peptide (1.25 µg/ml) diluted 1:1 with coupling buffer. Flow-through fractions per animal were pooled and analyzed by ELISA and as noted above.

The SyH7 and IB2 capture ELISA with biotin-labeled RT.^36^ Immulon 4HBX 96-well microtiter plates (ThermoFisher Scientific) were coated with SyH7 or IB2 MAbs (1 µg/ml in PBS) overnight at 4^o^C. The plates were blocked with goat serum (2%), washed, and then overlaid with a mixture of biotin-labeled RT and serial dilutions of Rhesus macaque competitor serum. The amount of biotin-RT used in the competition ELISA was equivalent to the EC_90_ for each capture MAb (100–150 ng/ml). The plates were washed and developed with streptavidin-HRP (1:1000; ThermoFisher Scientific) and TMB. The percent (%) inhibition of ricin binding was calculated from the optical density (OD) values as follows: 1−value OD_450_ (biotin-ricin + competitor Ab) ÷ value OD_450_ (biotin-ricin without competitor Ab) × 100.

### Statistical analysis

Statistical analysis was carried out with GraphPad Prism 8.2. The mean survival times after exposure to RT were compared by log-rank analysis of Kaplan–Meier survival curves. The statistical significance of the effects of ricin challenge and huPB10 intervention on cytokine/chemokine/growth factor levels were analyzed with two-way repeated measures ANOVAs in both serum and BAL, with the repeated measures being pre- and post-exposure status, and treatment group as the independent measure. Resulting p-values were corrected with the Benjamini, Krieger, and Yekutieli method to control false discovery rate. If a significant interaction effect was found by two-way ANOVA, Šidák’s multiple comparisons tests were performed to compare pre-challenge to post-challenge values for each group. Due to the low abundance of many of the inflammatory markers in serum and BAL fluids examined by Luminex, all analyses were performed on log_2_ transformed raw fluorescent intensity values to avoid the need to censor values.^37^ A P value less than 0.05 was considered significant. PCA analysis was performed using singular value decomposition with the R package FactoMineR.^38^ Friedman tests with post hoc Dunn’s multiple comparisons tests were used to compare pre-challenge levels of de novo anti-RT IgG with levels from post-challenge timepoints. The same procedure was used to compare pre- and post-challenge levels of epitope-specific MAb competition.

## Supplementary information

Supplementary Information

Supplementary Data 1

## Data Availability

All data generated or analyzed during this study are included in this published article (and its supplementary information files). All relevant data are available upon request from the authors.

## References

[1] Wolfe DN, Florence W, Bryant P (2013). Current biodefense vaccine programs and challenges. Hum. Vaccin Immunother..

[2] Zilinskas RA, Mauger P (2015). E-commerce and biological weapons nonproliferation: Online marketplaces challenge export controls to reduce the risk that rogue states or terrorists could acquire the capacity to produce biological weapons. EMBO Rep..

[3] Cieslak TJ (2018). Beyond the dirty dozen: a proposed methodology for assessing future bioweapon threats. Mil. Med..

[4] Sowa-Rogozinska, N., Sominka, H., Nowakowska-Golacka, J., Sandvig, K. & Slominska-Wojewodzka, M. Intracellular transport and cytotoxicity of the protein toxin ricin. *Toxins (Basel)* **11**, 10.3390/toxins11060350 (2019). 10.3390/toxins11060350 PMC6628406 31216687

[5] Endo Y, Mitsui K, Motizuki M, Tsurugi K (1987). The mechanism of action of ricin and related toxic lectins on eukaryotic ribosomes. The site and the characteristics of the modification in 28 S ribosomal RNA caused by the toxins. J. Biol. Chem..

[6] Endo Y, Tsurugi K (1987). RNA N-glycosidase activity of ricin A-chain. Mechanism of action of the toxic lectin ricin on eukaryotic ribosomes. J. Biol. Chem..

[7] Gal, Y. et al. Treatments for pulmonary ricin intoxication: current aspects and future prospects. *Toxins (Basel)* **9**, 10.3390/toxins9100311 (2017). 10.3390/toxins9100311 PMC5666358 28972558

[8] Pincus SH (2015). Clinical and pathological findings associated with aerosol exposure of macaques to ricin toxin. Toxins (Basel).

[9] Sapoznikov, A. et al. Early disruption of the alveolar-capillary barrier in a ricin-induced ARDS mouse model: neutrophil-dependent and -independent impairment of junction proteins. *Am. J. Physiol. Lung Cell Mol. Physiol.*10.1152/ajplung.00300.2018 (2018). 10.1152/ajplung.00300.2018 30382767

[10] Lindauer ML, Wong J, Iwakura Y, Magun BE (2009). Pulmonary inflammation triggered by ricin toxin requires macrophages and IL-1 signaling. J. Immunol..

[11] Roy, C. J. et al. Rescue of Rhesus macaques from the lethality of aerosolized ricin toxin. *JCI Insight* **4**, 10.1172/jci.insight.124771 (2019). 10.1172/jci.insight.124771 PMC6485354 30626745

[12] Wong J, Korcheva V, Jacoby DB, Magun B (2007). Intrapulmonary delivery of ricin at high dosage triggers a systemic inflammatory response and glomerular damage. Am. J. Pathol..

[13] Rong, Y., Westfall, J., Ehrbar, D., LaRocca, T. & Mantis, N. J. TRAIL (CD253) sensitizes human airway epithelial cells to toxin-induced cell death. *mSphere* **3**, 10.1128/mSphere.00399-18 (2018). 10.1128/mSphere.00399-18 PMC6158510 30258037

[14] Froude JW, Stiles B, Pelat T, Thullier P (2011). Antibodies for biodefense. MAbs.

[15] Van Slyke G (2016). Humanized monoclonal antibody that passively protects mice against systemic and intranasal ricin toxin challenge. Clin. Vaccin. Immunol..

[16] Kuo TT, Aveson VG (2011). Neonatal Fc receptor and IgG-based therapeutics. MAbs.

[17] Zalevsky J (2010). Enhanced antibody half-life improves in vivo activity. Nat. Biotechnol..

[18] Verreault, D. et al. Effective treatment of staphylococcal enterotoxin B (SEB) aerosol intoxication in Rhesus macaques using two parentally-administered high affinity monoclonal antibodies. *Antimicrob. Agents Chemother.*10.1128/AAC.02049-18 (2019). 10.1128/AAC.02049-18 PMC6496046 30782986

[19] Toth, R. T. I. et al. High-definition mapping of four spatially distinct neutralizing epitope clusters on RiVax, a candidate ricin toxin subunit vaccine. *Clin. Vaccine Immunol.* **24**, 10.1128/CVI.00237-17 (2017). 10.1128/CVI.00237-17 PMC5717194 29046307

[20] Roy CJ (2015). Thermostable ricin vaccine protects Rhesus macaques against aerosolized ricin: Epitope-specific neutralizing antibodies correlate with protection. Proc. Natl Acad. Sci. USA.

[21] Domachowske JB (2018). Safety, tolerability and pharmacokinetics of MEDI8897, an extended half-life single-dose respiratory syncytial virus prefusion f-targeting monoclonal antibody administered as a single dose to healthy preterm infants. Pediatr. Infect. Dis. J..

[22] Griffin, M. P. et al. Safety, tolerability, and pharmacokinetics of MEDI8897, the respiratory syncytial virus prefusion F-targeting monoclonal antibody with an extended half-life, in healthy adults. *Antimicrob. Agents Chemother*. **61**, 10.1128/AAC.01714-16 (2017). 10.1128/AAC.01714-16 PMC5328523 27956428

[23] Gaudinski MR (2018). Safety and pharmacokinetics of the Fc-modified HIV-1 human monoclonal antibody VRC01LS: A Phase 1 open-label clinical trial in healthy adults. PLoS Med..

[24] Yu, X. Q. et al. Safety, tolerability, and pharmacokinetics of MEDI4893, an investigational, extended-half-life, anti-staphylococcus aureus alpha-toxin human monoclonal antibody, in healthy adults. *Antimicrob. Agents Chemother*. **61**, 10.1128/AAC.01020-16 (2017). 10.1128/AAC.01020-16 PMC5192133 27795368

[25] Yang, B. et al. ZMappTM reinforces the airway mucosal barrier against ebola virus. *J. Infect. Dis.*, 10.1093/infdis/jiy230 (2018). 10.1093/infdis/jiy230 PMC6093450 29688496

[26] Yermakova A (2016). Neutralizing monoclonal antibodies against disparate epitopes on ricin toxin’s enzymatic subunit interfere with intracellular toxin transport. Sci. Rep..

[27] Hussell T, Bell TJ (2014). Alveolar macrophages: plasticity in a tissue-specific context. Nat. Rev. Immunol..

[28] Rong, Y. et al. An intranasally administered monoclonal antibody cocktail abrogates ricin toxin-induced pulmonary tissue damage and inflammation. *Hum. Vaccin Immunother*, 1–15, 10.1080/21645515.2019.1664243 (2019). 10.1080/21645515.2019.1664243 PMC7227631 31589555

[29] Worbs T, Hammerschmidt SI, Forster R (2017). Dendritic cell migration in health and disease. Nat. Rev. Immunol..

[30] DiLillo DJ, Ravetch JV (2015). Fc-Receptor interactions regulate both cytotoxic and immunomodulatory therapeutic antibody effector functions. Cancer Immunol. Res..

[31] Naranjo-Gomez M, Pelegrin M (2019). Vaccinal effect of HIV-1 antibody therapy. Curr. Opin. HIV AIDS.

[32] Schoofs T (2016). HIV-1 therapy with monoclonal antibody 3BNC117 elicits host immune responses against HIV-1. Science.

[33] Lemley PV, Wright DC (1992). Mice are actively immunized after passive monoclonal antibody prophylaxis and ricin toxin challenge. Immunology.

[34] Tate MK, Rico PJ, Roy CJ (2004). Comparative study of lung cytologic features in normal Rhesus (Macaca mulatta), cynomolgus (Macaca fasicularis), and African green (Chlorocebus aethiops) nonhuman primates by use of bronchoscopy. Comp. Med..

[35] O’Hara JM, Brey RN, Mantis NJ (2013). Comparative efficacy of two leading candidate ricin toxin a subunit vaccines in mice. Clin. Vaccin. Immunol..

[36] Westfall J (2018). Thermal stability and epitope integrity of a lyophilized ricin toxin subunit vaccine. Vaccine.

[37] Breen EJ, Tan W, Khan A (2016). The statistical value of raw fluorescence signal in luminex xMAP based multiplex immunoassays. Sci. Rep..

[38] Lê S, Josse J, Husson F (2008). FactoMineR: An R package for multivariate analysis. J. Stat. Softw..

