# Supplementary material for: Passive immunization with an extended half-life monoclonal antibody protects Rhesus macaques against aerosolized ricin toxin
Source: NPJ Vaccines. 2020 Feb 13;5:13. doi: 10.1038/s41541-020-0162-0 (PMC7018975; doi:10.1038/s41541-020-0162-0)
Supplement: Supplementary file 1 — Supplementary Information [file 41541_2020_162_MOESM1_ESM.pdf]

Figure S1

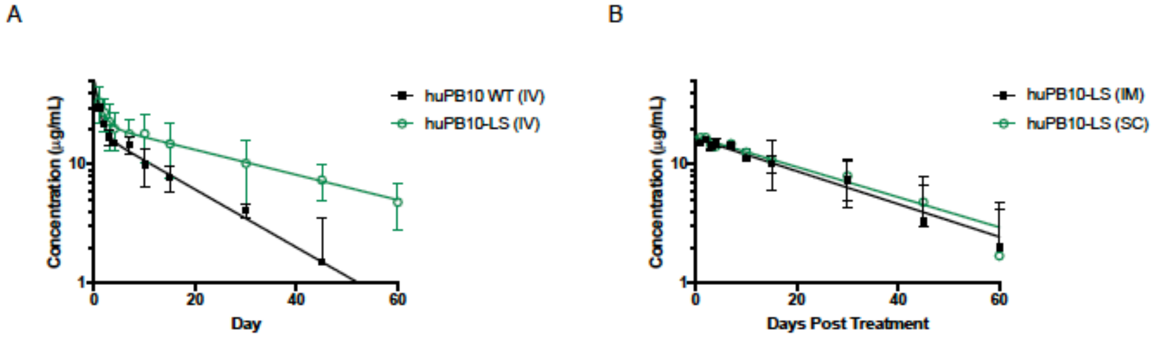

C.

Half-life of huPB10 IgG1 and IgG1-LS variant				
	**huPB10 WT (IV)**	**huPB10-LS (IV)**	**huPB10-LS (IM)**	**huPB10-LS (SC)**
**$t_{1/2}$ α (days)**	0.7	0.9		
**$t_{1/2}$ β (days)**	12.4	28.5	21.8	23.7
**AUC (µg/mL)xd**	328.7	682.6	433.8	477.1

Figure S1. PK analysis of huPB10-LS in Rhesus macaques following IV, IM and SC administration. Serum profiles (A,B) and PK parameters (C) of huPB10 and the hu-PB10-LS variants following (A) intravenous (IV) or (B) intramuscular (IM) or subcutaneous (SC) injections, as described in the Materials and Methods. The relative amounts of huPB10 and huPB10-LS in the serum samples were determined using a custom Octet-based assay. The absolute amounts of huPB10 and huPB10-LS in serum determined by the Octet assay were underestimated by ~3-fold, as determined by a quantitative capture ELISA (see Materials and Methods) .

Figure S2

A.

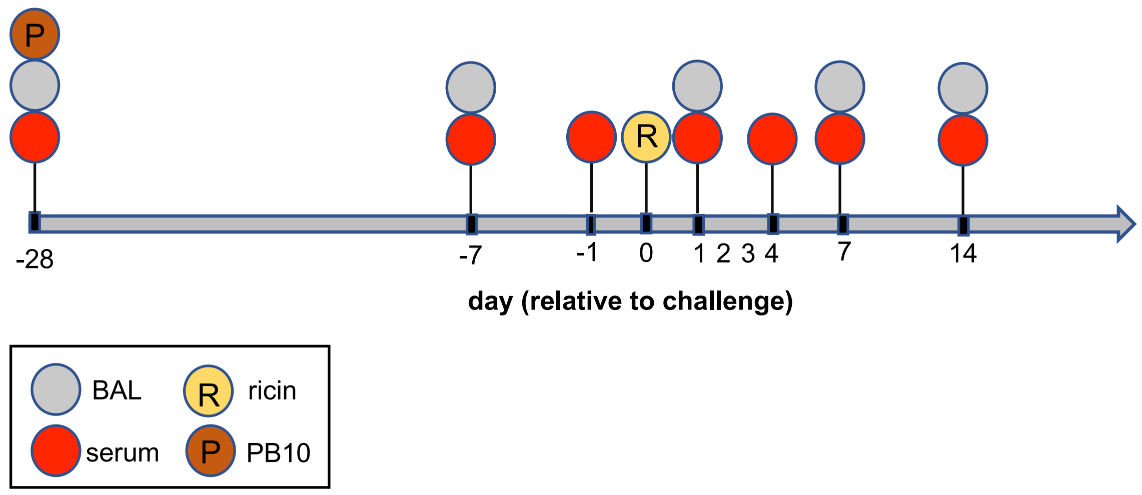

B.

date	day count	huPB10-LS (IV)	ricin AEROSOL challenge	Ab determination	BAL
6/25/2018	-28	X		2 x 1.1 ml	X (40 ml)
7/16/2018	-7			2 x 1.1 ml	X (40 ml)
7/23/2018	0		X	2 x 1.1 ml	
7/24/2018	1			2 x 1.1 ml	X (40 ml)
7/25/2018	2			2 x 1.1 ml	
7/26/2018	3			2 x 1.1 ml	
7/27/2018	4			2 x 1.1 ml	
7/30/2018	7			2 x 1.1 ml	X (40 ml)
8/06/2018	14			2 x 1.1 ml	X (40 ml)
8/13/2018	21	Necropsy			

Figure S2. Timeline and associated sampling schedule for experimental evaluation of huPB10-LS in the rhesus macaque. A. Experimental timeline with milestone biosampling, administration, and aerosol challenge. **B.** Sampling schedule with date and day count noted for biosampling, administration, aerosol challenge, and necropsy.

FigureS3

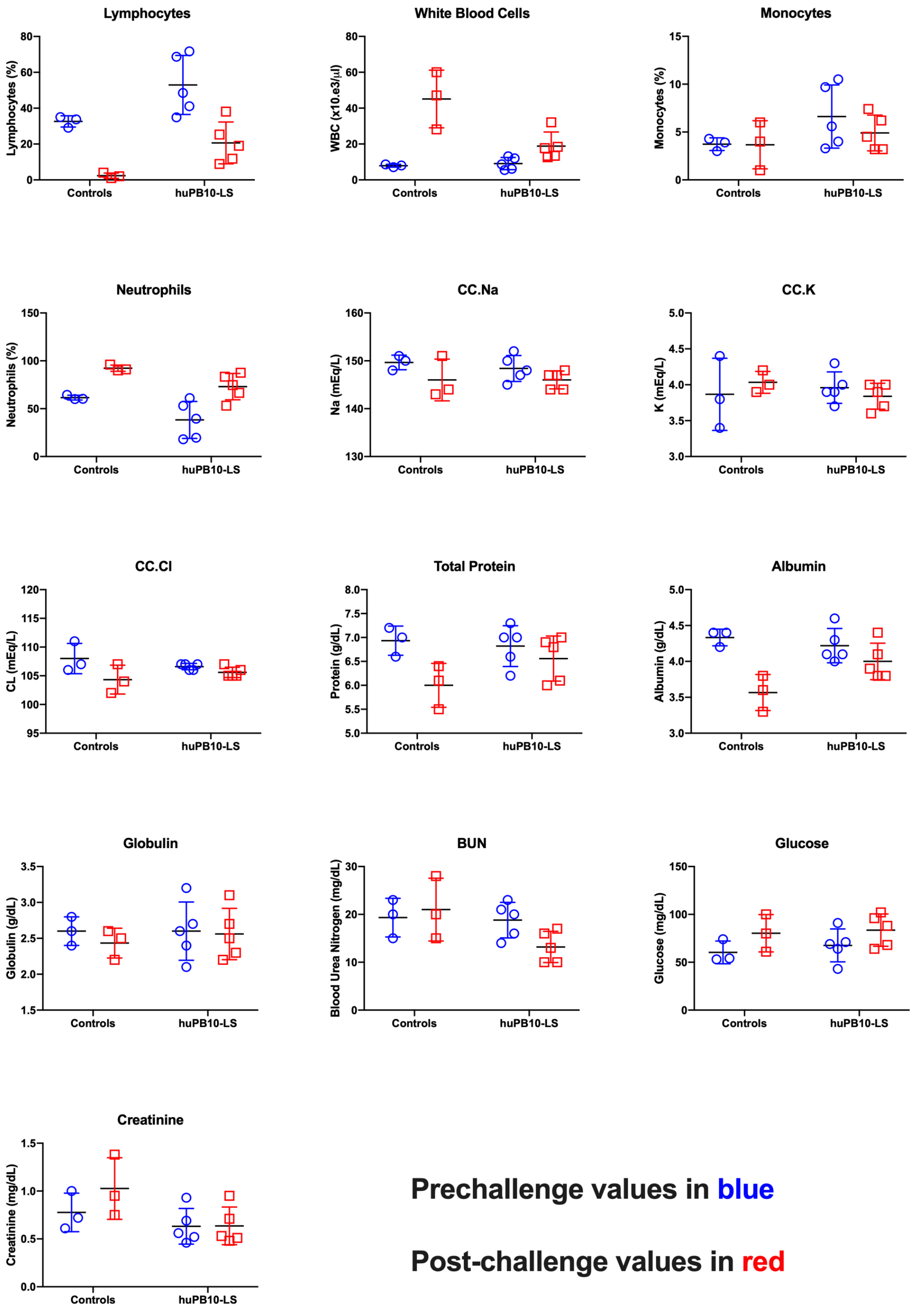

Figure S3. Changes in hematologic and serum chemistries in groups of Rhesus macaques before and after RT exposure. Blood was collected before (blue) and ~22 h after (red) RT exposure. For each panel, control animals are shown on left and huPB10-LS treated animals on right. Automated blood counts and chemistry analysis were performed using best clinical practices at the Tulane National Primate Research Center.

Figure S4

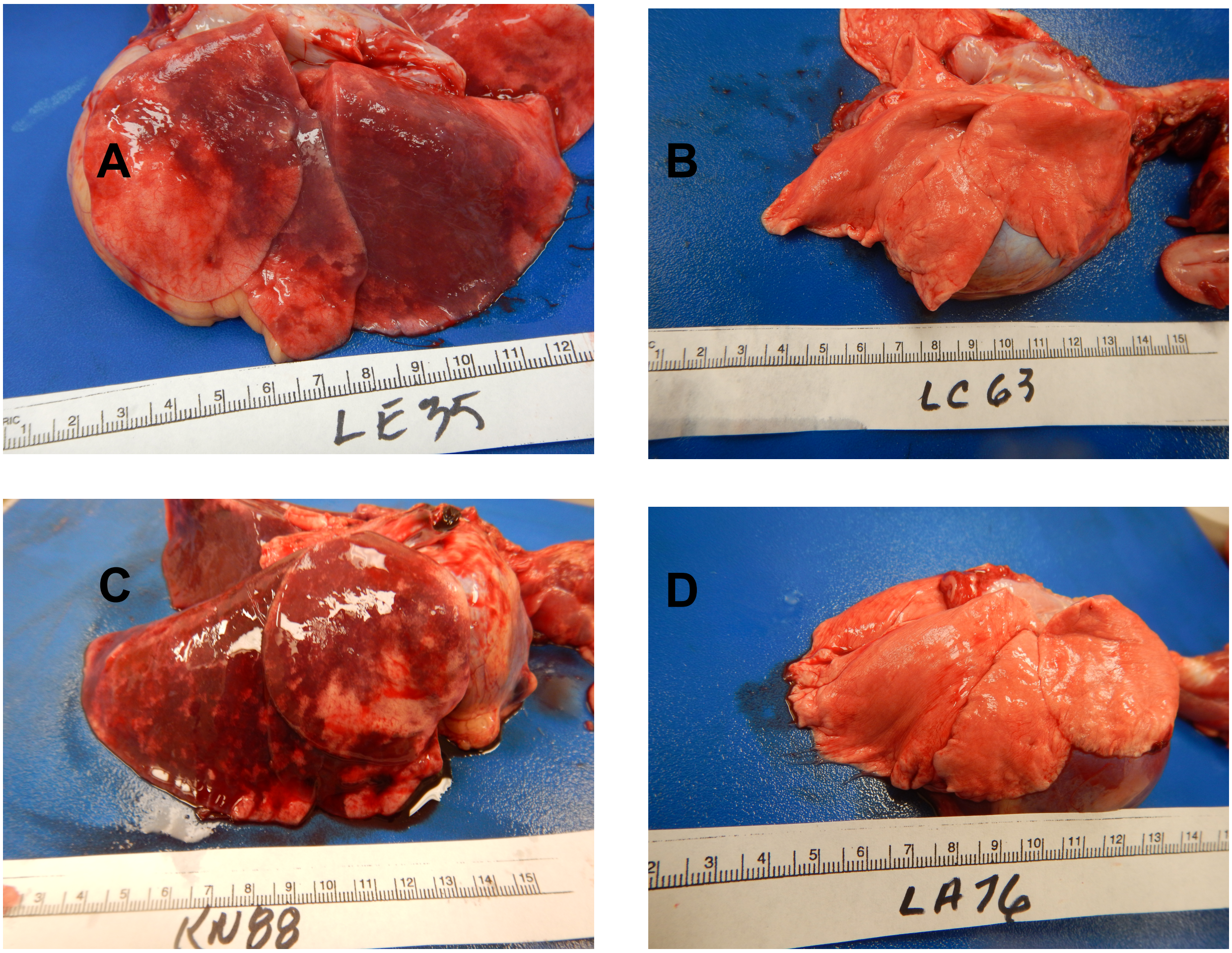

Figure S4. Gross lung pathology of control or huPB10-LS-treated animals challenged with aerosolized ricin toxin. Each alphanumeric character references the animal identification number, treatment received, and the following description, **A**. LE35, control (Ig121), hemorrhagic, enlarged, wet, and firm; **B**. LC63, huPB10-LS, no remarkable changes; **C.** KN88, control (Ig121), hemorrhagic, enlarged, wet, and firm; **D.** LA76, huPB10-LS, no remarkable changes.

Figure S5

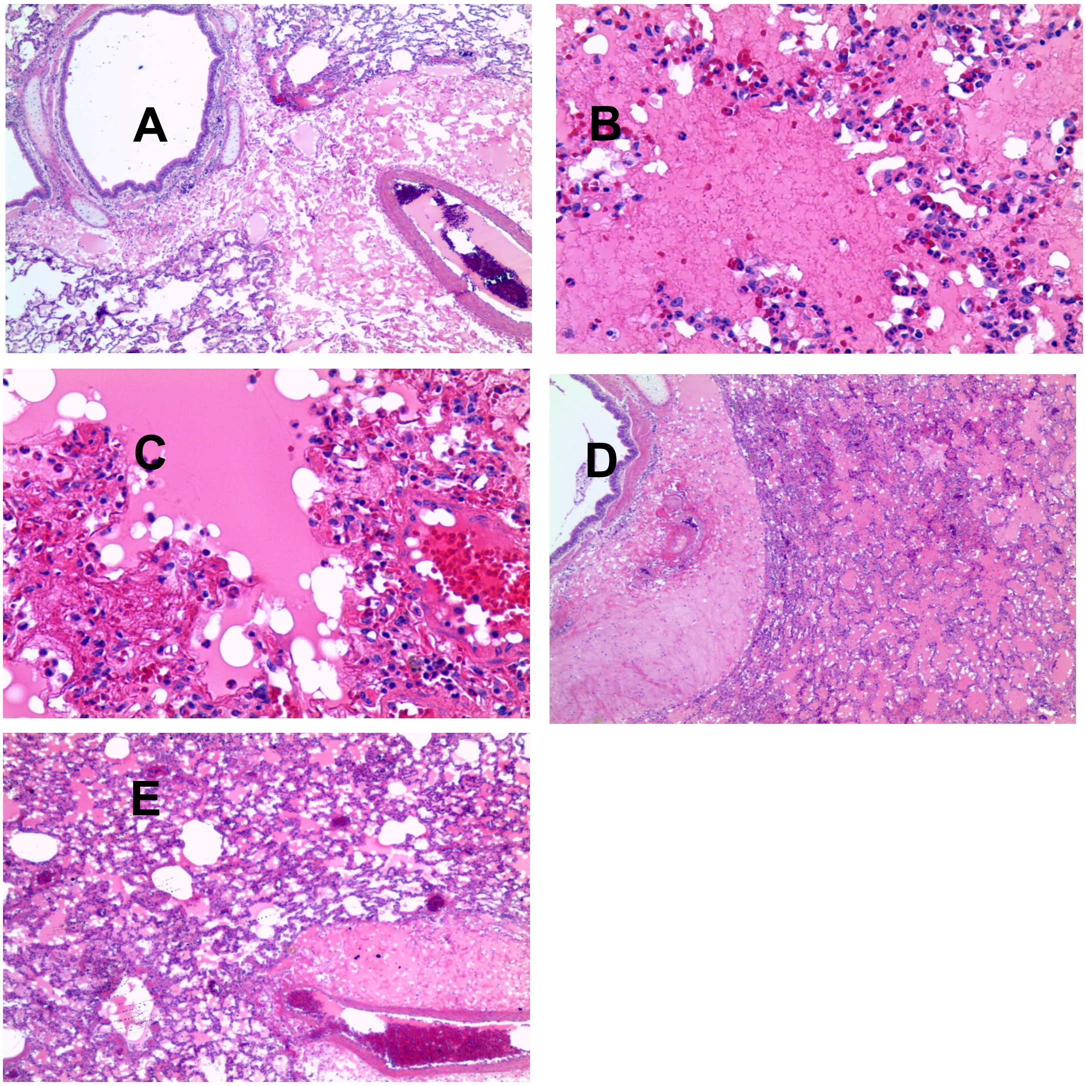

Figure S5. Lung histopathology of control animals challenged with aerosolized ricin toxin. Each alphanumeric character references the animal identification number, anatomic location, and the following description, **A**. LE35, lung, perivascular edema, 2x; **B**. KA06, lung, fibrin, edema, and acute inflammation of the the respiratory bronchiole 48hr PI, 20x; **C.** KN88, lung, acute inflammation, edema of the bronchiole, 20x; **D.** KA06, lung, edema inflammation, 48 hr 2x; **E.** KN88, lung, edema inflammation, 2x.

Figure S6

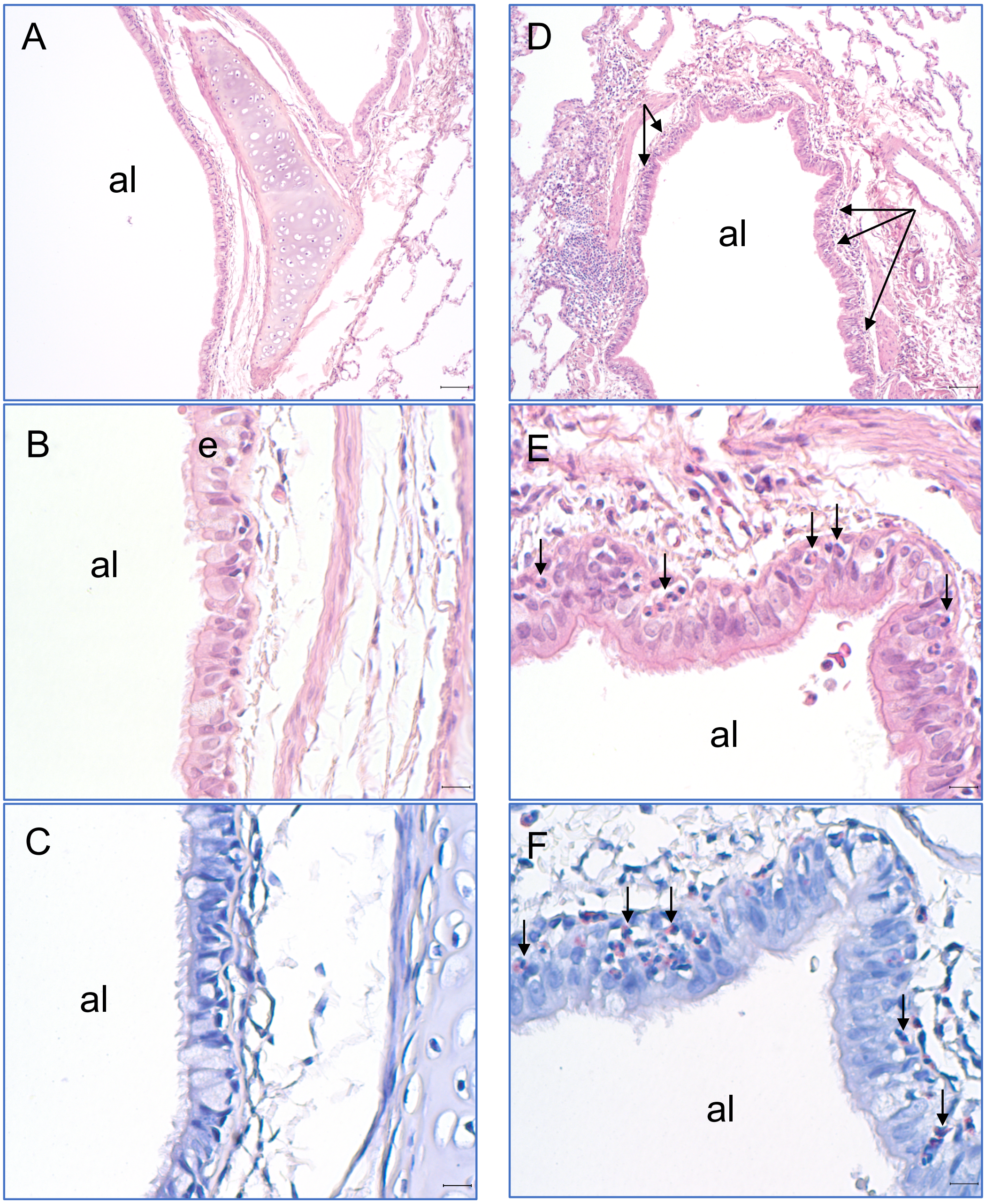

Figure S6. Histopathology (H&E) and immunohistochemistry (IHC) of huPB10-LS animals. Histopathological changes in most animals receiving PB10 treatment were within normal limits (A-C). The lumina of airways (al) were cleared and the epithelial surface (e) devoid of inflammatory cells (A-B). Most tissues were IHC negative for the eosinophil major basic protein (C). Minimal PMNs infiltration of airways epithelium was observed in 2 animals treated with PB10 (D-E). Inflammatory infiltrate consisted of comparable proportions of neutrophils and eosinophils (arrows), as highlighted in red by IHC (F). Bar = 50 μm (A and D), 10 μm all other images.

Figure S7

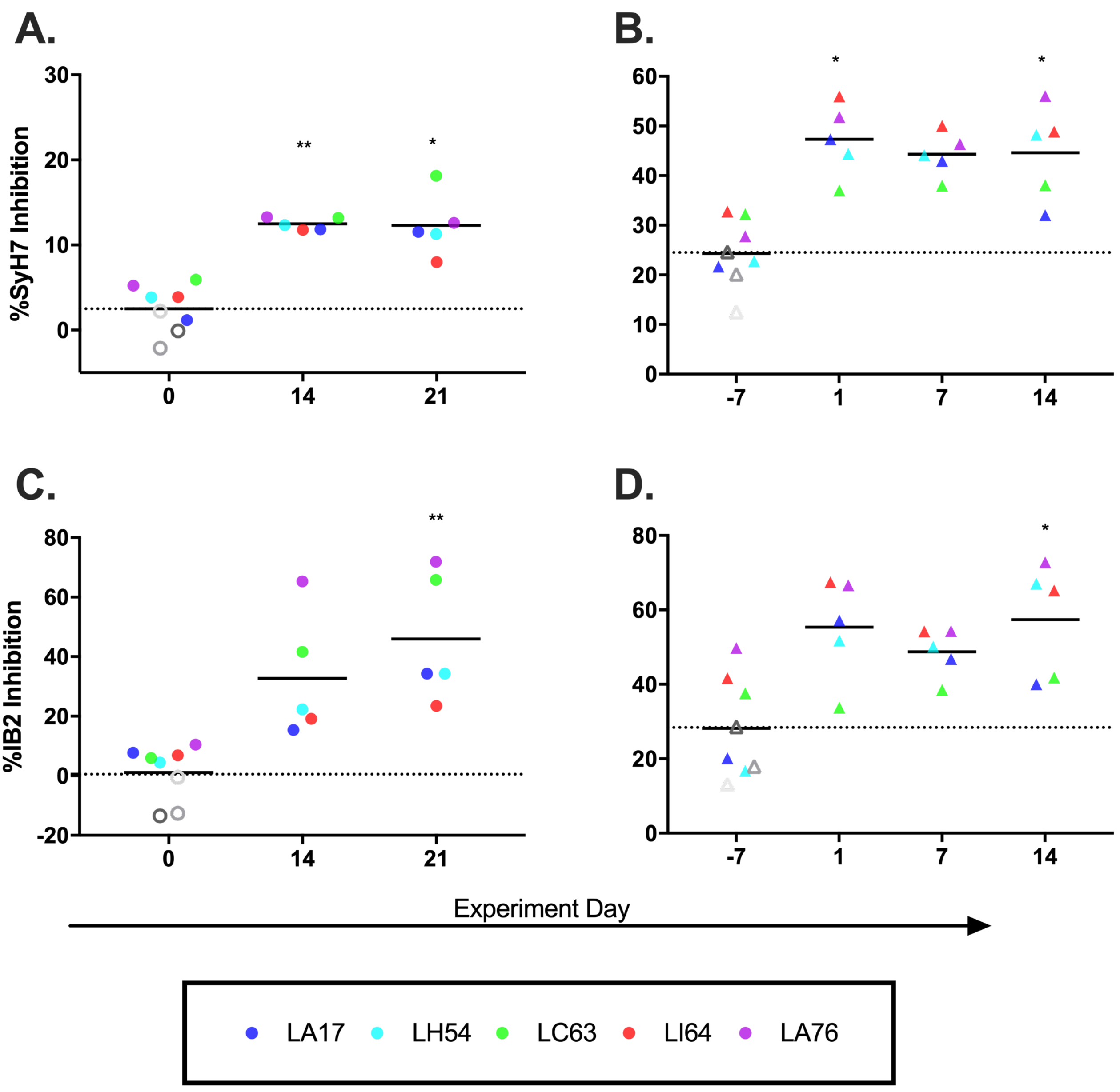

Figure S7-Appearance of de novo RTA-specific antibodies in serum and BAL fluids in huPB10-LS treated rhesus macaques following RT challenge. Serum samples (panels A, C) and BAL fluids (panels B, D) collected from experimental animals on indicated days (x-axes) were interrogated for the presences of antibodies against two spatially distinct immunodominant epitopes on RTA defined by SyH7 (left graphs) and IB2 (right graphs), as described in the text and in the legend to Figure 4. Panels A and C are the same as Figure 4 and reproduced here only for comparison with BAL fluids.

Figure S8

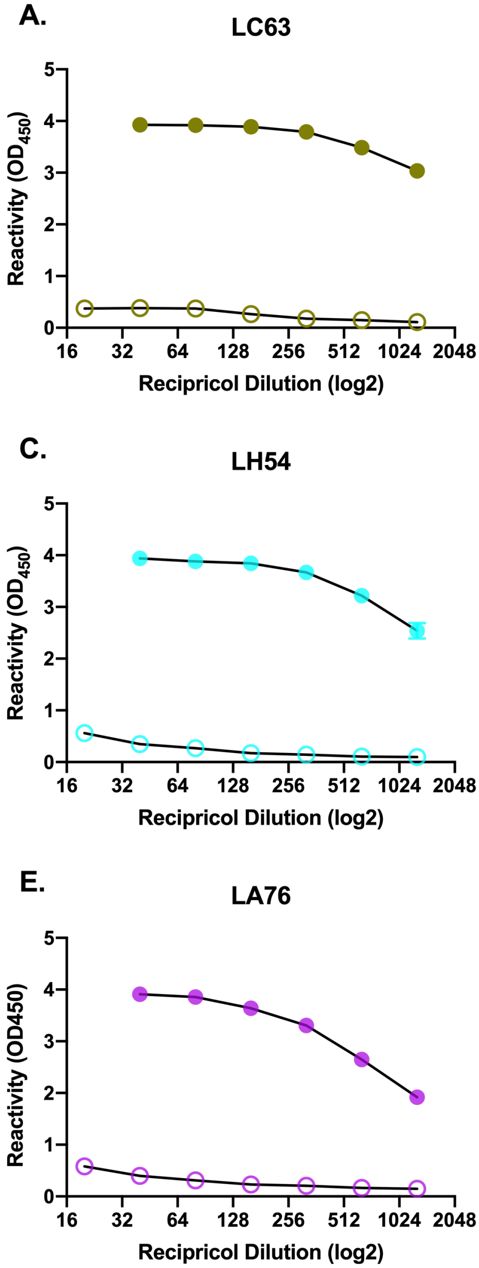

Figure S8. Efficacy of huPB10-LS depletion from NHP serum. Serum samples collected from experimental animals (A-E) on day 21 post-RT challenged were subjected to an E12-peptide affinity column to deplete them of residual huPB10-LS. The bulk serum (solid circle) or the E12 flow samples (open circles) were each serially diluted in PBS and evaluated for E12 reactivity by ELISA. Flow through samples were devoid of E12 reactivity when diluted more than 30-fold.

Dataset 1. Comprehensive statistical analysis of 29-plex Luminex array of serum and BALF samples.
